# Commensal-infected macrophages induce dedifferentiation and reprogramming of epithelial cells during colorectal carcinogenesis

**DOI:** 10.18632/oncotarget.22250

**Published:** 2017-11-01

**Authors:** Xingmin Wang, Yonghong Yang, Mark M. Huycke

**Affiliations:** ^1^ Department of Radiation Oncology, University of Oklahoma Health Sciences Center, Oklahoma City, OK 73104, USA; ^2^ The Muchmore Laboratories for Infectious Diseases Research, Oklahoma City VA Health Care System, Oklahoma City, OK 73104, USA; ^3^ Gansu Province Children's Hospital, Lanzhou, Gansu 730030, China; ^4^ Key Laboratory of Gastrointestinal Cancer, Lanzhou University Second Hospital, Lanzhou, Gansu 730030, China; ^5^ Department of Internal Medicine, University of Oklahoma Health Sciences Center, Oklahoma City, OK 73126, USA

**Keywords:** macrophage, bystander effect, Wnt/β-catenin, dedifferentiation, cancer stem cell

## Abstract

The colonic microbiome contributes to the initiation of colorectal cancer through poorly characterized mechanisms. We have shown that commensal-polarized macrophages induce gene mutation, chromosomal instability, and endogenous transformation through microbiome-induced bystander effects (MIBE). In this study we show that MIBE activates Wnt/β-catenin signaling and pluripotent transcription factors associated with dedifferentiation, reprogramming, and the development of colorectal cancer stem cells (CSCs). Exposure of murine primary colon epithelial cells (YAMC) to *Enterococcus faecalis*-infected macrophages increased Wnt3α expression while suppressing Wnt inhibitor factor 1 (Wif1). Wnt/β-catenin activation was confirmed by increased active β-catenin and Tcf4. *in vivo*, active β-catenin was evident in colon biopsies from *E. faecalis*-colonized *Il10* knockout mice compared to sham-colonized mice. This effect was mediated, in part, by 4-hydroxy-2-nonenal and tumor necrosis factor α. MIBE also activated pluripotent transcription factors c-Myc, Klf4, Oct4, and Sox2 in YAMC cells and colons from *E. faecalis*-colonized *Il10* knockout mice. These transcription factors are associated with cellular reprogramming, dedifferentiation, and induction of colorectal CSC progenitors. In support of this was an increase in the expression of Dclk1 and CD44, two colorectal CSC markers, in YAMC cells that were exposed to MIBE. Finally, compared to normal colon biopsies and hyperplastic polyps, DCLK1 expression increased in human tubular adenomas and invasive colorectal cancers. Blocking β-catenin/TCF4 signaling using FH535 and *CTNNB1*-specific small interfering RNA decreased DCLK1 expression in HCT116 human colon cancer cells. These findings provide mechanism for microbiome-induced colorectal cancer and identify new potential targets for colorectal cancer prevention.

## INTRODUCTION

Cancer stem cells (CSCs) play pivotal roles in major solid tumor heterogeneity, tumor growth and metastasis, and resistance to chemotherapy [1]. The cells of origin for CSCs remain controversial. Evidence suggests that CSCs may originate from normal adult stem cell populations that have acquired somatic mutations and undergone transformation. Quiescent adult stem cells are attractive targets for mutation because of their long life span. Persistence allows for the gradual accumulation of mutations that drive malignant transformation [2]. On the other hand, CSCs may originate from long-lived non-malignant non-stem cell progenitors or differentiated tumor cells through reprogramming and dedifferentiation [1, 3, 4]. As with many solid tumors, colorectal cancer is considered to be composed of small populations of self-renewing CSCs that divide and differentiate to generate tumor heterogeneity [5]. The relationship between CSCs in colorectal cancer and role of the human microbiome in promoting their development is, however, not fully understood.

In murine models of colorectal cancer deletions and/or mutations of the adenomatous polyposis coli gene (*Apc*) in intestinal stem cells are associated with cellular transformation and tumor formation. This suggests that these cells, or long-lived descendants, are cells of origin for CSCs [2]. In contrast, other studies have identified fully differentiated and long-lived tuft cells as cells of origin for colorectal CSCs [6–8]. In both scenarios, *Apc* mutations along with dysregulated Wnt/β-catenin signaling are drivers for the development of CSCs [6, 7]. Wnt/β-catenin regulates numerous processes in cellular differentiation and tissue homeostasis [9]. This signaling is also involved in CSC development in colorectal cancer [10, 11]. Several commensals such as *Bacteroides fragilis* and *Fusobacterium nucleatum* can activate Wnt/β-catenin signaling [12, 13]. Whether activation of β-catenin by these or other commensals contributes to the development of colorectal CSCs remains uncertain. Understanding early triggers for aberrant Wnt/β-catenin signaling, especially arising from the microbiome, will help decipher initiating events for colorectal cancer.

Inflammation is considered a major risk factor for colorectal cancer [14]. Part of this risk derives from NF-kB signaling and Wnt activation that induces reprogramming and dedifferentiation of epithelial cells into stem-cell-like cells [6]. We have shown that selected intestinal commensals can be pro-inflammatory by polarizing colon macrophages into an M1 state, thereby generating endogenous mutagens and inflammatory cytokines [15–20]. These factors lead to cellular proliferation, aneuploidy, chromosomal instability, and malignant transformation of epithelial cells. We term these events as microbiome-induced bystander effects (MIBE). They represent a novel mechanism by which commensals interact with innate immune cells to generate mutations and transformation leading to colorectal cancer [17].

One mediator for MIBE is 4-hydroxy-2-nonenal (4-HNE), a DNA mutagen and mitotic spindle inhibitor derived from the peroxidation of ω6 polyunsaturated fatty acids [16]. Tumor necrosis factor α (TNFα) activates Wnt/β-catenin signaling [20, 21] and also contributes to MIBE. In a murine model of MIBE where colon macrophages are depleted by liposomal clodronate, both colon inflammation and tumor formation were blocked [15]. In another study, MIBE resulted in the formation of multicellular spheroids and teratomas, malignant transformation of a primary colon epithelial cell, and enhanced expression of the stem/progenitor cell markers lymphocyte antigen 6 complex, locus A (Ly6A/E) and doublecortin like kinase 1 (Dclk1) [17]. These observations prompted us to investigate transcription factors and cellular signaling associated with MIBE as it reprograms, dedifferentiates, and transforms colon epithelial cells into CSCs.

In this study we found that MIBE activated Wnt/β-catenin signaling and multiple pluripotent transcription factors. These transcription factors were associated with the expression of CSC markers. *in vitro* studies confirmed 4-HNE and TNFα as independent drivers of these markers. Finally, we noted increased DCLK1 expression in association with Wnt/β-catenin signaling in human tubular adenomas and invasive colorectal cancers, but not normal colon tissue. These findings demonstrate that MIBE activates Wnt/β-catenin signaling and induces pluripotent transcription factors associated with dedifferentiation, reprogramming, and transformation of primary colon epithelial cells.

## RESULTS

### Commensal-infected macrophages activate Wnt/β-catenin signaling

**Figure 1:**
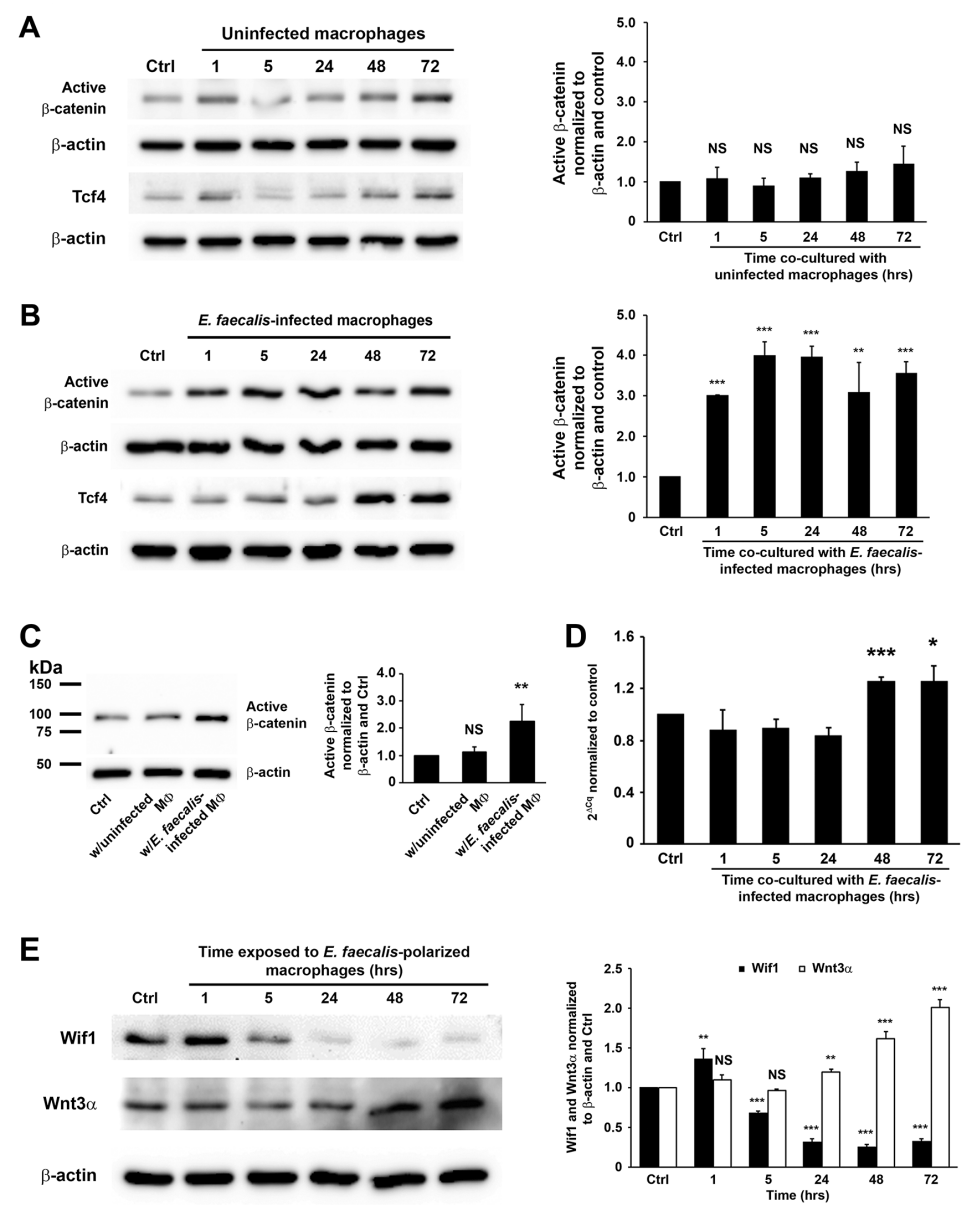
*E. faecalis*-infected macrophages activate Wnt/β-catenin signaling **(A)**, Active β-catenin (unphosphorylated) is not seen in YAMC cells exposed to uninfected macrophages (*left*) compared to untreated controls after normalization to β-actin (*right*). **(B)**, Exposure of YAMC cells to *E. faecalis*-infected macrophages increases active β-catenin (*left*) compared to untreated control after normalization to β-actin (*right*). Treatments also increase expression of Tcf4. **(C)**, Western blots (*left*) show increased active β-catenin in nuclear extracts from YAMC cells treated with *E. faecalis*-infected macrophages compared to untreated YAMC control and cells treated with uninfected macrophages after normalization to β-actin (*right*). **(D)**, qRT-PCR confirms increased *Ctnnb1* expression in cells exposed to *E. faecalis*-infected macrophages compared to untreated controls. **(E)**, Western blots show decreased expression of Wif1 in YAMC cells at 5 to 72 hrs following exposure to *E. faecalis*-infected macrophages. In contrast, Wnt3α increases in cells at 24 to 72 hrs following treatment. MФ, macrophage; NS, not significant; ^*^ *P* < 0.05, ^**^ *P* < 0.01, and ^***^ *P* < 0.001 compared to Ctrl. Data represent mean ± SD for 3 independent experiments.

To investigate activation of Wnt/β-catenin signaling by MIBE, murine primary colon epithelial cells (YAMC) were co-cultured with uninfected or *E. faecalis*-infected macrophages that were polarized to an M1 state by *E. faecalis* [15, 17]. As expected, exposure of YAMC cells to uninfected macrophages did not activate β-catenin (Figure 1A), although an increase of *Ctnnb1* expression was seen 48 hrs following exposure (Supplementary Figure 1A). In contrast, *E. faecalis*-infected macrophages increased active β-catenin, Tcf4 (a binding target for active β-catenin), and inactive p-Gsk3β (Ser^9^) 24 to 72 hrs following co-culture (Figure 1B and Supplementary Figure 2A). Notably, increased active β-catenin was seen in nuclear extracts from cells treated with *E. faecalis*-infected macrophages compared to untreated control and cells treated with uninfected macrophages (Figure 1C), supporting nuclear translocation induced by MIBE. In addition, *E. faecalis*-infected macrophages increased *Ctnnb1* expression in YAMC cells (Figure 1D). Of note, *E. coli*-infected macrophages also increased *Ctnnb1* expression, active β-catenin, and Tcf4 in YAMC cells (Supplementary Figures 1B and 3). To explore mechanisms for Wnt/β-catenin activation, we assessed Wnt3α and Wif1 as known modulators of Wnt signaling [22, 23]. Wnt3α increased at 24 to 72 hrs following co-culture of YAMC cells with *E. faecalis*-infected macrophages (Figure 1E). In contrast, Wif1 decreased at 5 hrs and remained suppressed throughout the experiment. These results show that MIBE activates canonical Wnt/β-catenin signaling by suppressing Wif1 and activating Wnt3α.

### MIBE activates β-catenin in vivo

**Figure 2:**
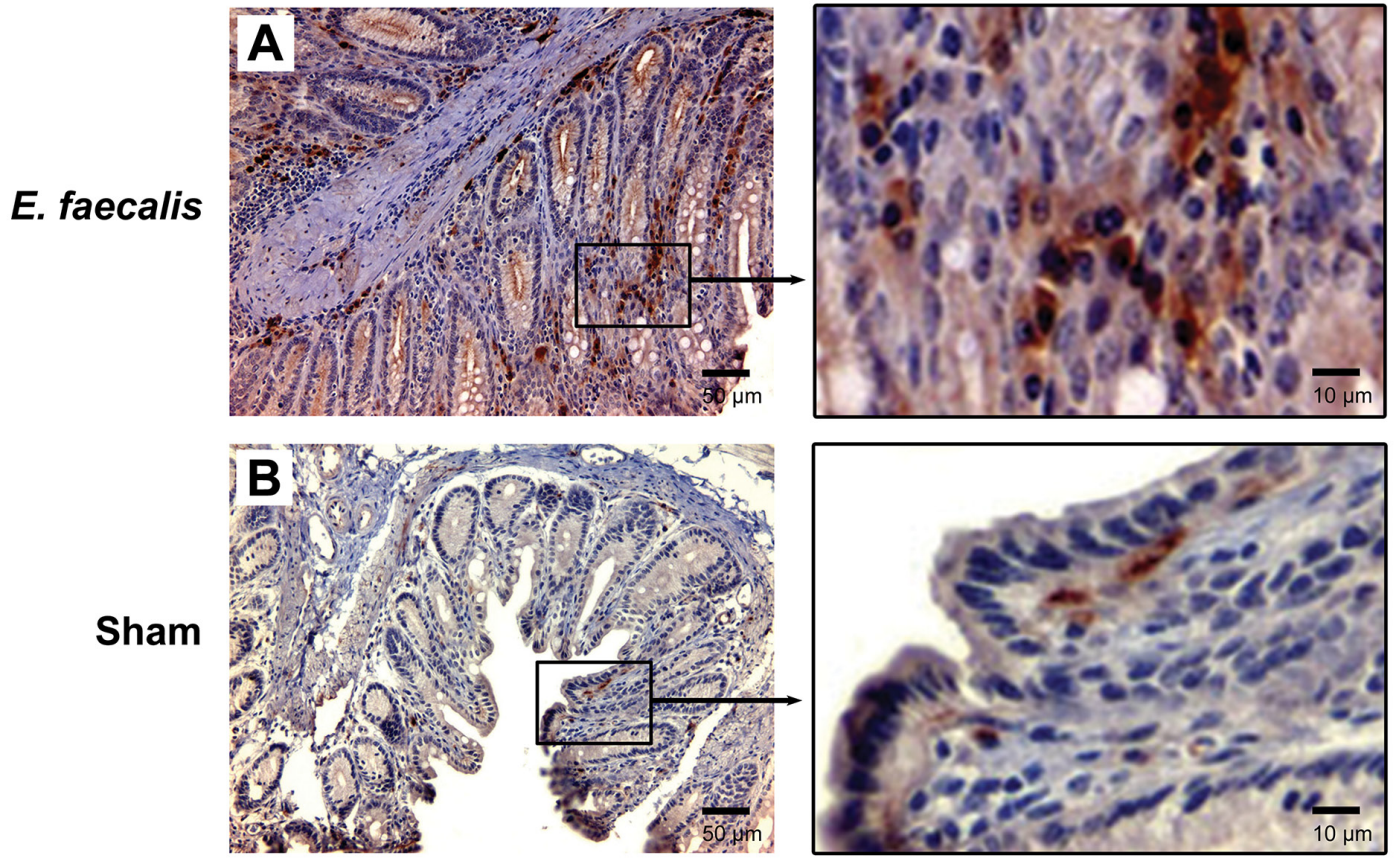
*E. faecalis* colonization activates Wnt/β-catenin signaling in IL-10 knockout mice **(A)**, Representative microphotographs for immunohistochemical staining show positivity for active β-catenin in both stromal and epithelial cells from colon biopsies for *Il10*^-/-^ mice colonized with *E. faecalis* for 9 months (*left*, 20 x). Positive staining is present in both cytoplasm and nuclei (*right*, 100 x). **(B)**, no staining is seen in colon biopsies from sham-colonized mice (*left*, 20 x; *right*, 100x).

*E. faecalis*-colonized *Il10*^-/-^ mice—a model for MIBE leading to colorectal cancer—developed colitis and colorectal cancer after 9 months of colonization [16]. These mice maintained colonization with *E. faecalis* at ~10^9^ colony-forming units per gram feces during entire experiment [16]. When colon biopsies from these mice were examined for active β-catenin, strong staining was found in stromal and epithelial cells compared to sham-colonized mice (Figure 2), supporting β-catenin activation by MIBE *in vivo*.

### 4-HNE and TNFα activate Wnt/β-catenin

**Figure 3:**
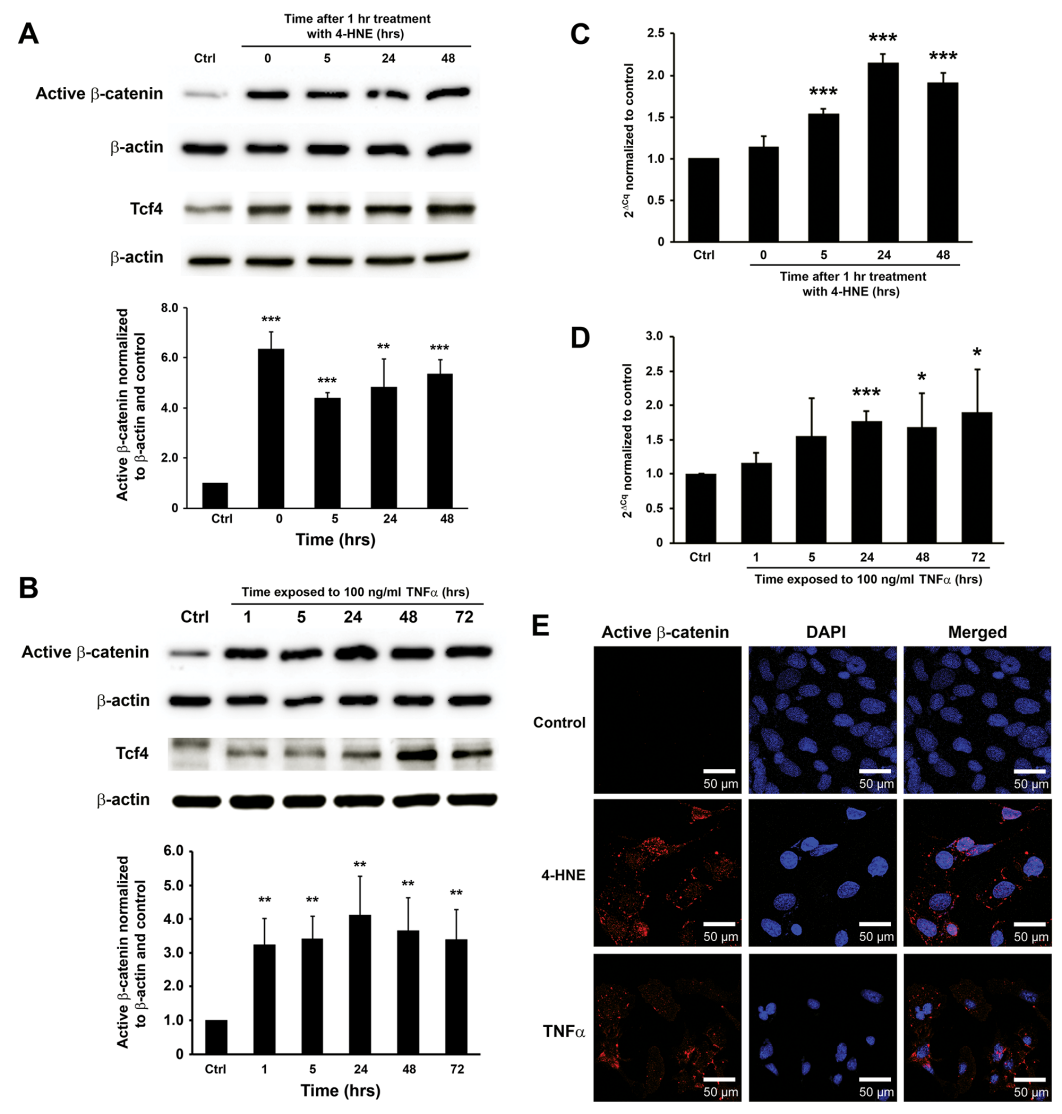
4-HNE and TNFα mediate MIBE-induced Wnt/β-catenin activation **(A)**, Western blots show increased active β-catenin and Tcf4 in YAMC cells following 1 hr treatment with 1 μM 4-HNE. **(B)**, TNFα similarly induces increased active β-catenin and Tcf4 in YAMC cells. **(C)**, qRT-PCR shows increased *Ctnnb1* expression after 4-HNE treatment. **(D)**, Increased expression of *Ctnnb1* is also confirmed in YAMC cells treated with TNFα for 24 to 72 hrs. **(E)**, Immunofluorescent staining for active β-catenin shows stabilization and nuclear translocation of β-catenin in YAMC cells 5 hrs following treatment with 4-HNE (*middle*) and TNFα (*bottom*) compared to untreated control (*top*). Of note, non-specific staining of nuclear antigens by anti-active-β-catenin antibody is not evident in controls (*top left*). DAPI, 4’,6-diamidino-2-phenylindole. NS, not significant; ^*^ *P* < 0.05 and ^***^ *P* < 0.001 compared to Ctrl. Data represent mean ± SD for 3 independent experiments.

Previous studies by us showed that 4-HNE and TNFα were mediators for MIBE [16, 20, 24]. To determine whether these mediators could directly activate Wnt/β-catenin signaling, YAMC cells were treated with purified 4-HNE and TNFα. Western blots showed increased active β-catenin, Tcf4, and inactive p-Gsk3β after a 1 hr-treatment with 4-HNE (Figure 3A and Supplementary Figure 2B). TNFα similarly induced active β-catenin, Tcf4, and inactive p-Gsk3β (Figure 3B and Supplementary Figure 2C). qRT-PCR confirmed increased gene expression of *Ctnnb1* at 5 to 48 hrs following 4-HNE treatment (Figure 3C). Increased expression of *Ctnnb1* was also noted in cells treated with TNFα at 24 to 72 hrs (Figure 3D). Finally, staining for active β-catenin showed nuclear translocation for YAMC cells treated with 4-HNE or TNFα compared to untreated controls (Figure 3E). These findings show that these MIBE mediators can each independently induce Wnt/β-catenin signaling in colon epithelial cells.

### MIBE induces pluripotent transcription factors

**Figure 4:**
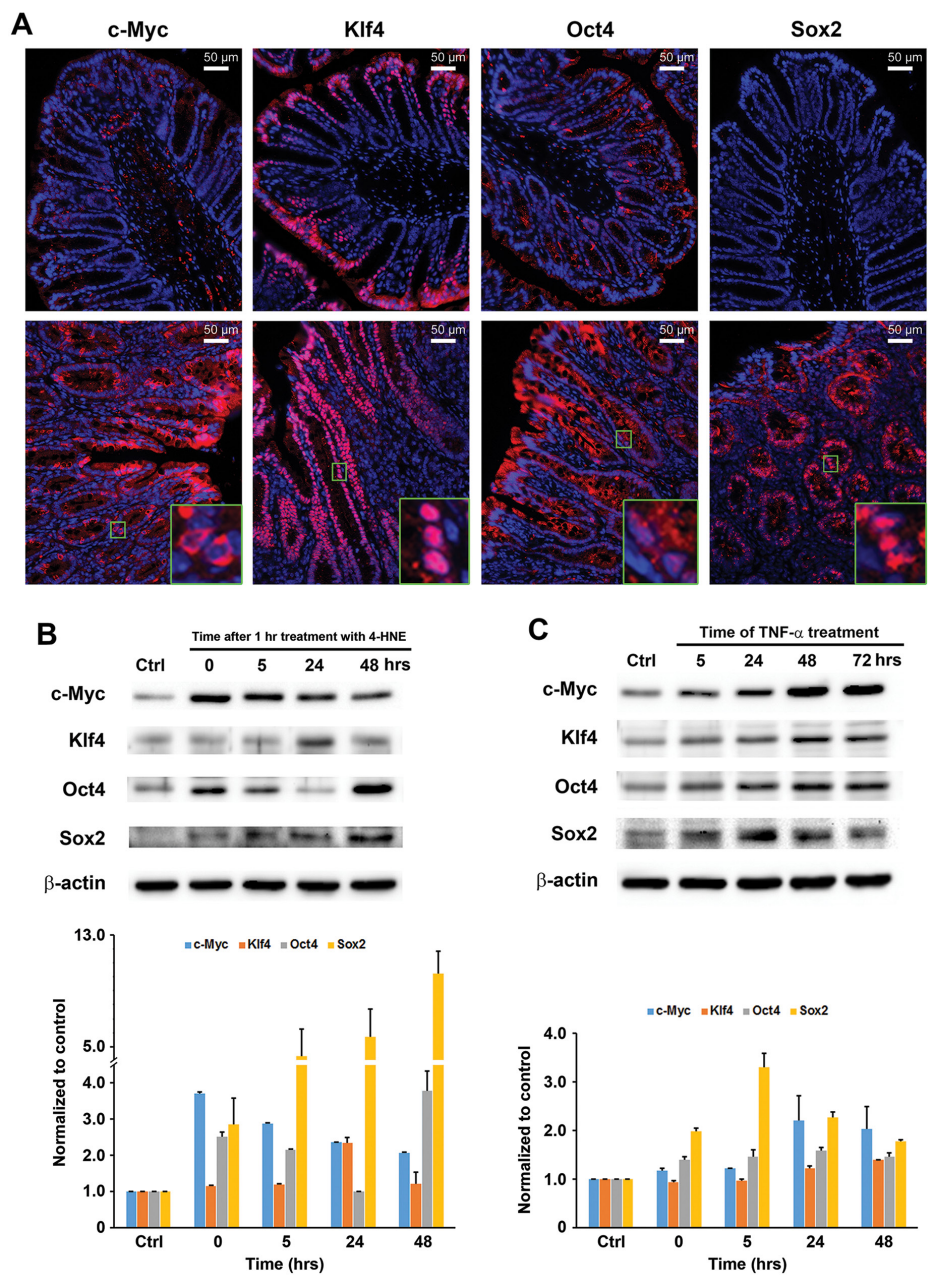
MIBE activates pluripotent transcription factors **(A)**, Immunofluorescent staining shows increased c-Myc, Klf4, Oct4, and Sox2 in epithelial cells for colon biopsies from *E. faecalis*-colonized *Il10*^-/-^ mice (*lower panels*, 20x) compared to sham-colonized mice (*upper panels*, 20x). Insets (100x) show differing patterns for these factors. **(B)**, Western blots show increased c-Myc, Klf4, Oct4, and Sox2 expression in YAMC cells treated with 4-HNE for 1 hr (*upper*) after normalized to untreated control (*lower*). **(C)**, Similarly, TNFα induces c-Myc, Klf4, Oct4, and Sox2 in these cells (*upper*). *Lower* panel, normalization of Western blots with β-actin and untreated control. All data represent mean ± SD for 3 independent experiments.

We recently showed that commensal-infected macrophages induced progenitor and stem cell markers including Ly6A/E and Dclk1 in colon epithelial cells [17]. To further explore how MIBE reprograms colon epithelial cells, we measured expression of Yamanaka factors that induce pluripotent stem cells: c-Myc, Klf4, Oct3/4, and Sox2 [25]. We stained colon biopsies from *E. faecalis*-colonized *Il10*^-/-^ mice for these factors and noted increased expression in colon epithelial cells for each compared to biopsies from sham-colonized mice that otherwise showed no colitis or cancer (Figure 4A). Furthermore, there was increased expression of these same transcription factors in YAMC cells following treatment with 4-HNE or TNFα compared to untreated controls (Figures 4B and 4C). These results show that colon inflammation and MIBE mediators induce transcription factors associated with cellular dedifferentiation, reprogramming, and pluripotency.

### MIBE induces expression of colorectal CSC markers

**Figure 5:**
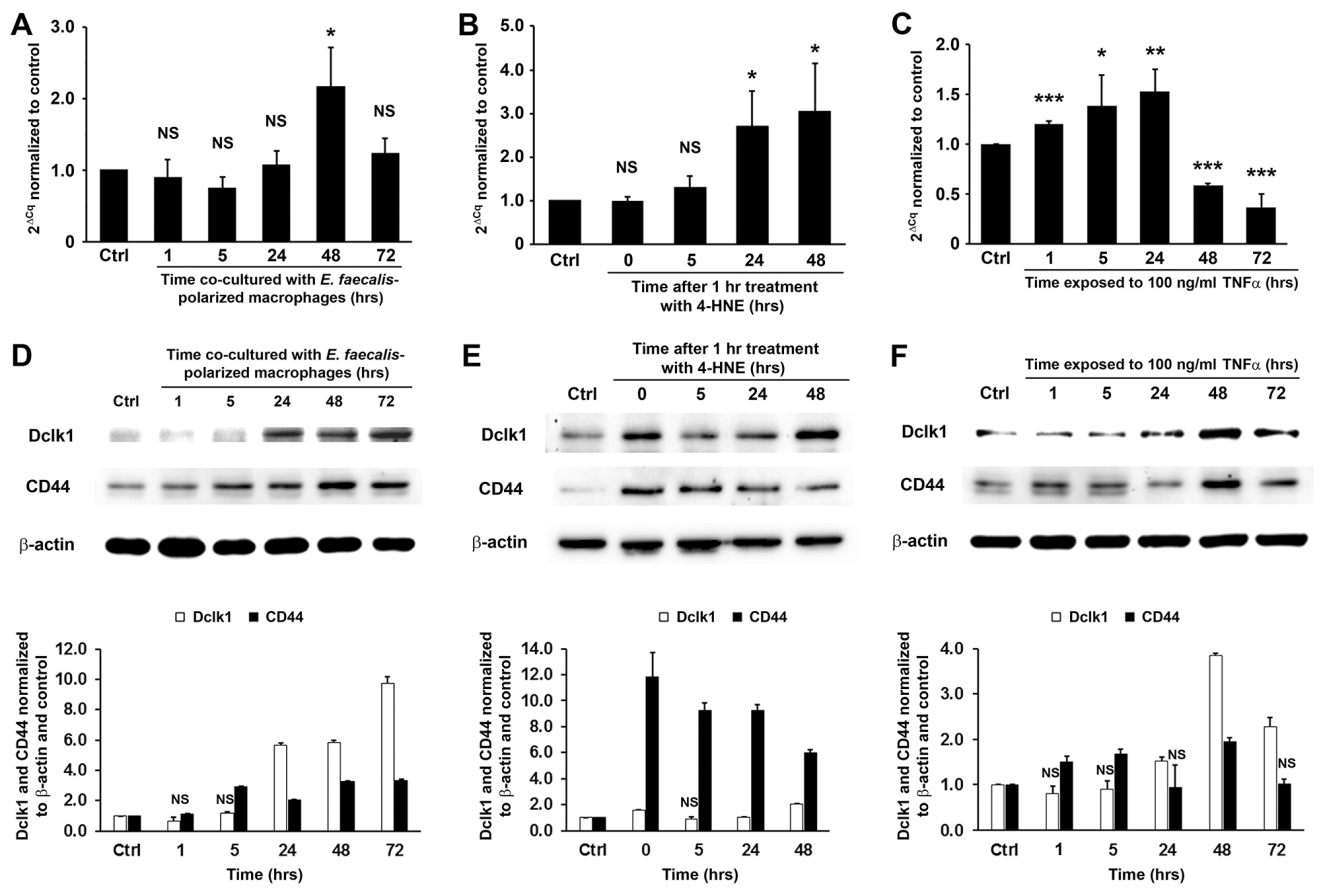
MIBE-induced expression of colon CSC markers **(A-C)**, qRT-PCR shows increased *Dclk1* expression in YAMC cells following treatments by *E. faecalis*-infected macrophages (A), 4-HNE (B), and TNFα (C). NS, not significant; ^*^ *P* < 0.05, ^**^ *P* < 0.01, and ^***^ *P* < 0.001 compared to Ctrl. **(D-F)**, Western blots confirm increased expression of Dclk1 and CD44, two colorectal CSC markers (*upper panels*), in YAMC cells treated with *E. faecalis*-infected macrophages (D), 4-HNE (E), and TNFα (F). *Lower panels*: normalization of Dclk1 and CD44 with β-actin and untreated controls (Ctrl). NS, not significant. All other comparisons are statistically significant at *P* < 0.01. All data represent mean ± SD for 3 independent experiments.

Dclk1, a colorectal CSC marker [7, 8], is strongly expressed in *E. faecalis*-triggered colorectal cancer and allograft tumors derived from YAMC cells treated by commensal-infected macrophages or purified 4-HNE [17]. To determine whether MIBE induces this CSC marker, we measured *Dclk1* expression in YAMC cells exposed to *E. faecalis*-infected macrophages, 4-HNE, or TNFα. In each instance, mRNA for Dclk1 increased (Figure 5A-5C). Western blots confirmed increased levels of Dclk1 protein (Figures 5D-5F). Finally, CD44, a downstream product of Wnt/β-catenin signaling and colorectal CSC marker [26], also showed increased expression in treated YAMC cells (Figure 5D-5F). These findings indicate that CSC markers associated with cellular reprogramming are regulated by MIBE.

### Wnt/β-catenin regulates DCLK1 expression in human colorectal cancer

**Figure 6:**
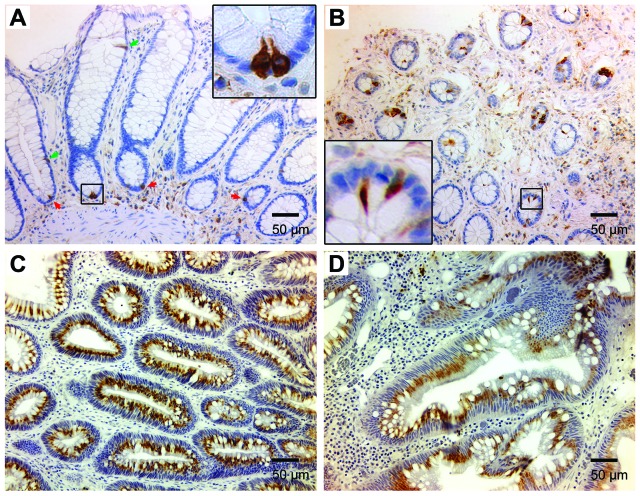
DCLK1 expression is increased in human colon adenomas and colorectal cancer **(A)**, Immunohistochemical staining for DCLK1 shows rare DCLK1^+^ cells in normal human colon at the bottom (*red arrows*) and upper portion (*green arrows*) of colon crypts (20x). Inset (100x) shows staining pattern for DCLK1^+^ cells at the crypt bottom. **(B)**, DCLK1^+^ cells are seen in hyperplastic polyps (20x). Inset (100x) shows a different staining pattern. **(C)** and **(D)**, DCLK1 staining is markedly increased for tubular adenomas (*C*) and invasive colorectal carcinomas (D).

To investigate DCLK1 expression in human colorectal cancer, biopsies from tubular adenomas and invasive cancers were immunohistochemically stained for DCLK1, and compared to staining for normal tissue and non-premalignant hyperplastic polyps. Staining showed rare DCLK1^+^ cells at the bottom and upper portions of crypts in normal human colon biopsies (Figure 6A). Although the number of DCLK1^+^ cells slightly increased in hyperplastic polyps, the staining pattern was similar to normal colon tissue (Figure 6B). In contrast, staining for DCLK1 in biopsies of tubular adenomas and invasive cancers was significantly increased compared to staining of normal and hyperplastic tissue (Figure 6C and 6D). DCLK1 staining in adenomas and cancers showed differing patterns compared to staining for normal and hyperplastic tissue. These findings were consistent with prior observations in *Il10*^-/-^ mice [17].

**Figure 7:**
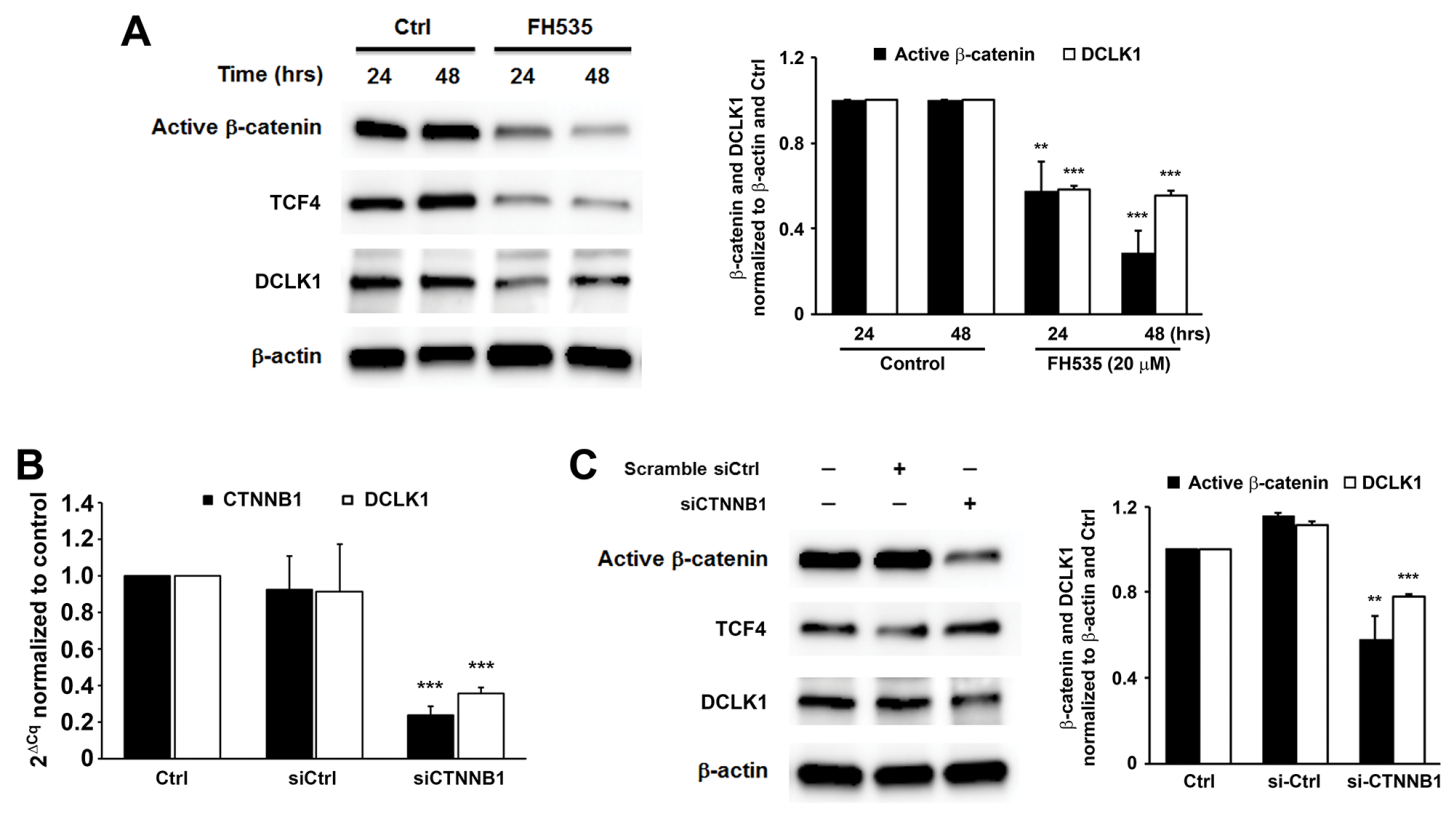
Wnt/β-catenin regulates DCLK1 expression in human colorectal cancer **(A)**, Inhibition of β-catenin/TCF4 by FH535 decreases DCLK1 expression in HCT116 colon cancer cells. **(B)**, qRT-PCR shows significantly decreased expression of *DCLK1* in *CTNNB1*-silenced HCT116 cells compared to controls. **(C)**, Western blots demonstrate that silencing *CTNNB1* in these cells has no effect on TCF4 expression but decreases DCLK1 expression. ^**^ *P* < 0.01 and ^***^ *P* < 0.001 compared to Ctrl. All data represent mean ± SD for 3 independent experiments.

To assess the role of β-catenin signaling in DCLK1 expression during colorectal carcinogenesis, we blocked this pathway in HCT116 cells using FH535, an inhibitor of β-catenin/Tcf4. Western blots showed 43% and 71% decreased β-catenin in treated cells at 24 and 48 hrs, respectively, compared to untreated controls (Figure 7A, *P* < 0.01 and *P* < 0.001, respectively). Similarly, FH535 reduced TCF4 expression by 68% and 83% at 24 and 48 hrs post-treatment, respectively (*P* < 0.001). This resulted in 42% and 44% decreased DCLK1 expression at 24 and 48 hrs, respectively (Figure 7A). To assess potential off-target effects for FH535, we silenced *CTNNB1* in HCT116 cells using human *CTNNB1*-specific siRNA and measured DCLK1 expression. *CTNNB1* expression was decreased by 76% at 24 hrs following transfection with *CTNNB1* siRNA compared to controls (*P* < 0.001). Partial silencing of *CTNNB1* resulted in a 61% decrease in *DCLK1* expression for cells transfected with *CTNNB1* siRNA compared to non-targeting siRNA (*P* < 0.001, Figure 7B). Western blots confirmed a 30% reduction in DCLK1 protein compared to cells treated with non-targeting siRNA (Figure 7C, *P* < 0.001). These results indicate that MIBE-induced Wnt/β-catenin signaling induces DCLK1 expression.

## DISCUSSION

Our findings show that MIBE activates Wnt/β-catenin signaling. This was associated with the induction of pluripotent transcription factors and CSC markers. These observations help link the human microbiome to dedifferentiation, reprogramming, and malignant transformation of primary colon epithelial cells. In a prior study, MIBE was found to efficiently transform primary colon epithelial cells into invasive carcinomas with the concomitant induction of stem/progenitor cell markers [17]. These findings along with results from this report support MIBE as a novel mechanism in which commensal-triggered innate immune responses promote endogenous mutagenesis and the development of CSCs from primary colon epithelial cells [24]. The theory predicts that abnormal triggering of colon macrophages by commensals, such as *E. faecalis*, leads to long-term polarization despite their otherwise known anergy. The result would be production of diffusible mediators that create a tissue microenvironment with low-level mutagenesis along with signals for reprogramming epithelial cells toward stemness. A progression toward malignant transformation would be expected.

**Figure 8:**
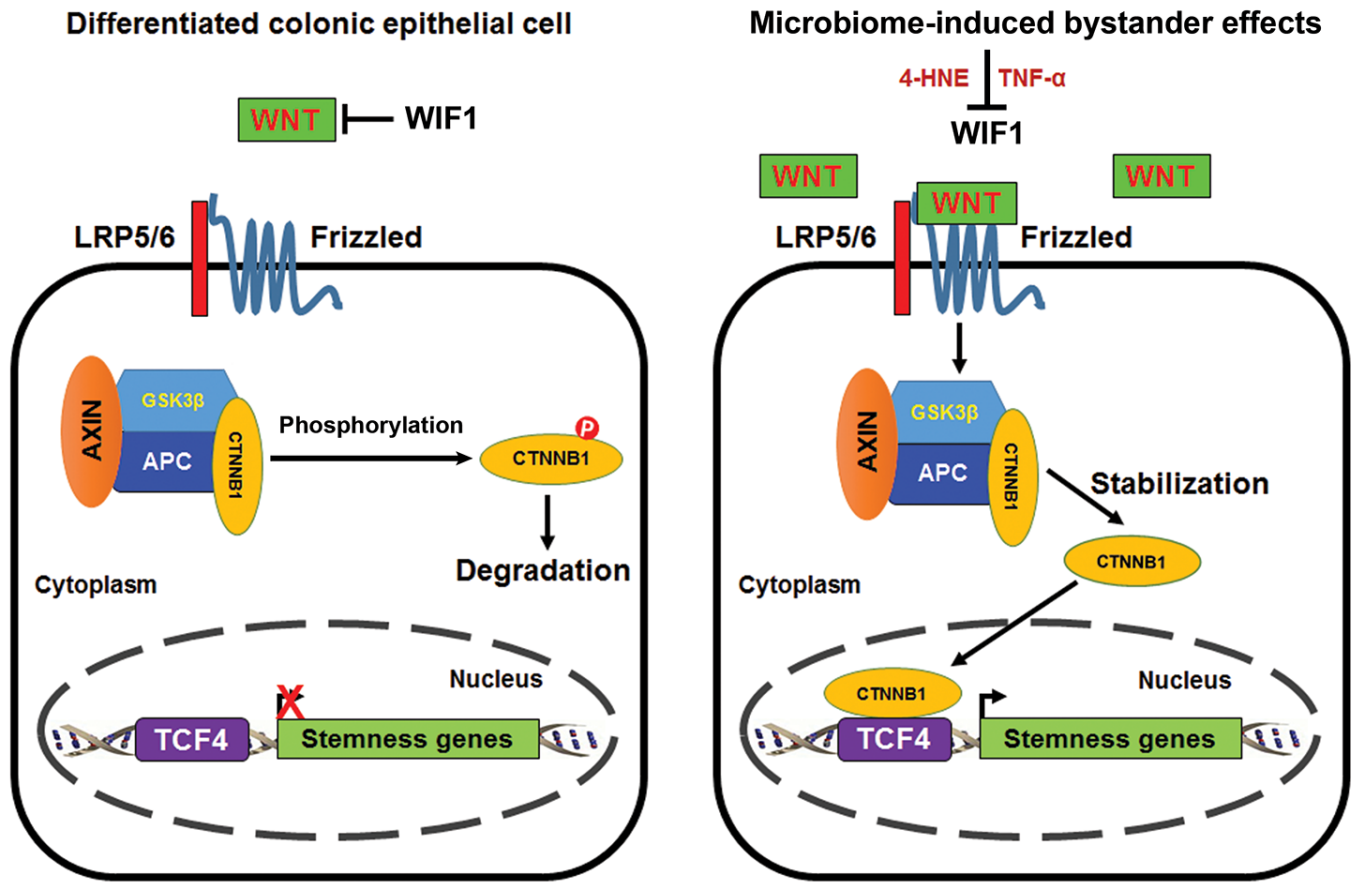
Proposed scheme for MIBE-induced activation of canonical Wnt/β-catenin signaling leading to cellular reprogramming and dedifferentiation Wnt/β-catenin signaling is inactive in normal differentiated colon epithelial cells due to the restraining mechanisms imposed on WNT by WIF1. Phosphorylated β-catenin is degraded by ubiquitin-mediated proteolysis (*left*). Commensal-infected macrophages produce diffusible mediators such as 4-HNE and TNFα that activate canonical Wnt/β-catenin signaling in colon epithelial cells. These mediators for MIBE act in concert by suppressing WIF1 and inducing WNT expression. MIBE leads to the nuclear translocation of active β-catenin where it binds TCF4 and induces pluripotent transcription factors. The final result is cellular reprogramming, dedifferentiation, and induction of CSC markers (*right*).

Wnt/β-catenin signaling occurs during normal stem cell development and tissue homeostasis [27], and also in association with many cancers including colorectal cancer [10]. In normal colon epithelial cells, where basal levels of Wnt are restrained by WIF1, β-catenin is constantly phosphorylated and degraded through proteasome-mediated ubiquitination (Figure 8, *left*). We found that 4-HNE and TNFα, two mediators of MIBE, each activated Wnt/β-catenin. This likely occurred by inducing Wnt3α and suppressing WIF1 suggesting post-translational activation of β-catenin. In addition, oxidative stress created by 4-HNE could also induce COX-2 in target cells. This enzyme produces 4-HNE as a byproduct of catalysis and the result would be a feedforward mechanism for further production and β-catenin activation [28]. Similar feedforward mechanisms may occur through TNFα. The subject merits further investigation.

Nuclear β-catenin binds TCF4 and induces pluripotent transcription factors and CSC markers (Figure 8, *right*). These findings were consistent with Wnt activation driving CSC development [6, 29]. Of note, a delay in Tcf4 expression was observed in cells exposed to MIBE. This may reflect posttranscriptional activation of Tcf4 by MIBE in advance of gene expression [30]. Sfrp2 is another Wnt suppressor that is down-regulated by promoter methylation in colorectal cancer [31]. We previously observed a sharp decrease in *Sfrp2* expression in primary colon epithelial cells transformed by MIBE or purified 4-HNE [17, 24]. Although we did not explore the possibility of MIBE-induced suppression of Sfrp2 in this study, our prior observations indicate that it may also contribute to Wnt/β-catenin activation.

4-HNE and TNFα contribute to the initiation of colorectal cancer as diffusible mediators of MIBE [16, 20]. In this study, each activated Wnt/β-catenin signaling and induced dedifferentiation of colon epithelial cells. Their mechanisms of action, however, are distinct. 4-HNE causes mutations, disrupts mitotic spindles, and induces tetraploidy and aneuploidy to generate chromosomal instability [16]. Although increased concentrations of this reactive aldehyde resulted in dedifferentiation of human pulmonary fibroblasts [32], its ability to induce dedifferentiation and stemness had not been previously reported. Covey et al. reported that 4-HNE induced p-Gsk3β (Ser^9^) to activate β-catenin in a fashion similar to that for YAMC cells [33]. Because Gsk3β is involved in several signaling pathways including Wnt/β-catenin and Nrf2 pathways [34], it remains unclear whether transiently increased p-Gsk3β is responsible for MIBE-induced β-catenin activation. In contrast, TNFα promotes inflammation and carcinogenesis by activating NF-κB and increasing the expression of the anti-apoptotic protein netrin-1 [20, 35]. TNFα can also cause DNA damage and mutations [36]. Interestingly, Cosín-Roger et al. found that macrophages polarized to the M2, but not M1 state secreted Wnt components that activated Wnt/β-catenin signaling and expression of downstream gene targets c-Myc and Lgr5 in colon epithelial cells [37, 38]. In this study, we show that 4-HNE and TNFα produced by M1-polarized macrophages are mediators that can activate Wnt/β-catenin signaling and potentially induce dedifferentiation of colon epithelial cells as a novel mechanism for commensal-initiated colorectal cancer.

Inflammatory signaling through NF-κB helps stabilize β-catenin and facilitates bidirectional conversion of intestinal epithelial cells into stem-cell-like physiology through dedifferentiation [6]. This is believed to be a precursor for tumorigenesis. For neurons, prostate cancer, and melanoma, TNFα-induced activation of NF-κB has been shown to help dedifferentiate somatic cells into cells with stem-cell-like properties that assist in repairing epithelial injury [39–42]. Although we did not test the combination of 4-HNE and TNFα *in vitro*, synergistic effects could be anticipated since TNFα induces COX-2 and this enzyme is a source for 4-HNE [28]. Indeed, when these mediators are produced in combination by MIBE, the result is rapid mutagenesis, dedifferentiation, reprogramming, and malignant transformation of target cells [17].

Seminal work by Takahashi and Nakayama showed that somatic cells can be reprogrammed to pluripotent stem cells by only four transcription factors: c-Myc, Klf4, Oct3/4, and SOX2 [25]. Each of these are regulated by Wnt/β-catenin signaling and were up-regulated in primary colon epithelial cells in our study [43–45]. These findings suggest that MIBE helps drive cellular dedifferentiation and reprogramming of non-stem colon epithelial cells as initial steps in the progression of these cells toward CSCs. Notably, the transcription factors c-Myc and Oct4 immediately increased within 1 hr of treatment by 4-HNE. c-Myc is an early response gene [46] and its induction by 4-HNE is consistent with its activation of other early response genes such as c-Jun and c-Fos [34]. Finally, we note that 4-HNE induced Oct4 expression in a bimodal fashion. While we did not explore this further, it may be due to positive feedback of the Oct4-Nanog-Tet1 network involving DNA demethylation of the Oct4 promoter sequence [47].

Teratoma formation is the gold standard for induced pluripotency [48]. In prior work, when YAMC cells were repetitively exposed to *E. faecalis*-infected macrophages or 4-HNE [17], anaplastic carcinomas rapidly developed. These experiments confirm the induction of pluripotency by MIBE with subsequent generation of CSCs.

An alternate approach to assess induced pluripotency involves measuring specific factors involved in dedifferentiation, *e.g.*, Oct-4, Nanog, and Sox2 [48]. Both *in vitro* and *in vivo* data showed that c-Myc, Klf4, Oct4, and SOX2 were each induced by MIBE. Although Nanog is a marker for pluripotent stem cells, we did not assess this transcription factor since is not required for somatic pluripotency in mice [49]. In our prior work, gene expression analysis of malignant clones created by MIBE identified altered expression of stromal cell-derived factor 1, insulin-like growth factor 2, and pleiotrophin [17, 24]. Loss of these growth factors likely enhanced dedifferentiation because, along with ephrin B1, they promote the differentiation of embryonic stem cells [50]. Finally, the findings from this study provide additional evidence for cellular dedifferentiation and reprogramming by MIBE. This work is also consistent with the increased expression of CSC markers Dclk1 and CD44, and progenitor stem cell marker Ly6A/E, as previously reported by us [7, 8, 17, 26, 51]. Taken together, these data strongly support MIBE as a potent mechanism for somatic cell dedifferentiation, reprogramming, and CSC development in colorectal cancer.

Two distinct models describe the morphogenesis of colorectal cancer: a “bottom-up” theory for CSCs arising from normal stem cells at the base of crypts [52], and a “top-down” hypothesis for CSCs arising from the upper sections of crypts with downward spread replacing normal crypt structures and stem cells [53]. The development of colorectal CSCs can be easily rationalized in a “bottom-up” concept with cells of origin arising from crypt stem cells at the base [54]. However, if colorectal CSCs were to arise through MIBE-induced dedifferentiation and reprogramming, then the cells of origin for CSCs could be differentiated colon epithelial cells that might occur at any position in the crypt—top, middle, or bottom. An MIBE theory for CSC development satisfies both “top-down” and “bottom-up” models.

Little is known about mechanisms that may potentially defend against MIBE. Glutathione *S*-transferases (GSTs) metabolize a wide range of electrophilic carcinogens [55]. 4-HNE, in particular, is inactivated by GST alpha 4 [56]. Induction of this enzyme may protect epithelial cells that are targeted by MIBE. GST alpha 4 is normally expressed in the colon and shows increased expression during inflammation. We found levels of Gsta4 were increased in colon macrophages from *Il10*^-/-^ mice colonized with *E. faecalis* for only 2 weeks [34]. Other potential defenses against MIBE include host polymorphisms to attenuate TNFα signaling through the Tnfrsf1a (Tnfr1) receptors on epithelial cells, promote inhibitory signals to maintain macrophages in a state of anergy, or interfere with epigenome remodeling by transcription factors involved in dedifferentiation. Much work is needed to better understand host defenses against MIBE and how these defenses vary based on epithelial cell location within the crypt.

Finally, we chose *E. faecalis* as a model microorganism for MIBE because *E. faecalis* produces extracellular superoxide [57]. This oxidative phenotype is not essential for inducing colitis in *Il10*^-/-^ mice but contributes to colorectal cancer development [16]. Nonetheless, other selected intestinal commensals can induce similar effects and trigger MIBE. For example, *E. coli*-infected macrophages also generate 4-HNE [16] and, as shown in this study, activate β-catenin. Investigation of other commensals that trigger MIBE will further our understanding of the links between the intestinal microbiome and transformational events leading to colorectal CSCs.

In summary, MIBE activates Wnt/β-catenin signaling in primary colon epithelial cells. Associated with this finding was an enhanced expression of transcription factors involved in induced pluripotency and colorectal CSC markers. These results provide evidence that selected members of the intestinal microbiome can drive colorectal carcinogenesis through dedifferentiation and reprogramming and should help expand strategies for targeting CSCs.

## MATERIALS AND METHODS

### Cell lines, bacteria, and chemicals

Murine primary colon epithelial cells (YAMC; Ludwig Institute for Cancer Research, New York, NY, USA), murine macrophages (RAW264.7; American Type Culture Collection, Manassas, VA, USA), and human colon cancer cells (HCT116; American Type Culture Collection) were grown as previously described [19]. *E. faecalis* OG1RF and *Escherichia coli* DH5α were grown overnight in brain-heart infusion (BD, NJ, USA) and Luria-Bertani broth (BD), respectively, at 37°C and washed with phosphate buffered saline (PBS) prior to infecting macrophages [18]. FH535 was purchased from Santa Cruz Biotechnology (Dallas, TX, USA). 4-HNE was purified by HPLC as previously described [16]. Murine TNFα was purchased from Cell Signaling Technology (Danvers, MA, USA).

### Infection of macrophages and treatment of YAMC cells

Infection of macrophages with *E. faecalis* and *E. coli* was performed as previously described [18]. RAW264.7 macrophages were grown overnight in antibiotic-free DMEM medium at 37°C and cell number determined by counting cells on duplicate plates. Overnight cultures of *E. faecalis* OG1RF and *E. coli* DH5α were centrifuged, washed with sterile PBS, and colony-forming units determined. Macrophages were treated with *E. faecalis* OG1RF and *E. coli* DH5α at a multiplicity of infection (MOI) of 1,000 and 100, respectively, in antibiotic-free medium for 1 hr at 37°C. As previously described, cells were washed with sterile PBS and numbers counted prior to use in a Transwell dual-chamber co-culture system (Corning, NY, USA) with macrophages growing on an insert and separated from YAMC cells by a membrane with 0.4 μM pores [18]. YAMC cells were co-cultured with macrophages in antibiotic-containing RPMI 1640/DMEM medium (1:1) at 33°C prior to collecting cellular RNA or protein at each time point.

For treatments with 4-HNE or TNFα, YAMC cells (5 × 10^5^) were grown overnight in 6-well plates and treated with purified 1 μM 4-HNE for 1 hr or 100 ng/ml murine TNFα for the duration of experiments at 33°C. Cells were washed and RNA or protein extracted at each time point.

### Animal study and human samples

Animal studies were approved by the University of Oklahoma Health Sciences Center and Oklahoma City VA Health Care System animal care and use committees. Specific pathogen-free *Il10^-/-^* mice (The Jackson Laboratory, Bar Harbor, ME, USA) were colonized with *E. faecalis* OG1RFSS (n = 7) or PBS sham (n = 7) as previously described [16]. Mice were necropsied after 9 months of colonization and colons fixed in 10% formalin. Normal human colon tissue arrays were purchased from US Biomax (Rockville, MD, USA). De-identified tissue blocks for human colon biopsies including hyperplastic colons (n = 30), tubular adenomas (n = 30), and invasive colorectal carcinomas (n = 30) were collected at the Oklahoma City VA Health Care System under a study approved by the University of Oklahoma Health Sciences Center Institutional Review Board and local Research and Development Committee.

### Real-time quantitative reverse transcriptase PCR (qRT-PCR)

RNA isolation and qRT-PCR were performed as previously described [34]. Primers for real-time qPCR of *CTNNB1*/*Ctnnb1*, *DCLK1*/*Dclk1*, and *ACTB*/*Actb* (Supplementary Table 1) were synthesized and purchased from Integrated DNA Technologies (Coralville, IA, USA). Complementary DNA (cDNA) was synthesized using TaqMan^®^ Reverse Transcription Reagents (Life Technologies, Grand Island, NY, USA) per manufacturer's instruction. qRT-PCR was carried out on the CFX96 Real-Time System (Bio-Rad, Hercules, CA, USA) using SYBR Premix Ex Taq II (Clontech Laboratories, Mountain View, CA, USA) at 95°C for 30 sec followed by 40 cycles at 95°C for 5 sec and 60°C for 30 sec. Expression of *CTNNB1*/*Ctnnb1* and *DCLK1*/*Dclk1* were normalized to ACTB/Actb and relative gene expression calculated by control.

### Separation of cytoplasmic and nuclear protein

YAMC cells were exposed to untreated macrophages or *E. faecalis*-infected macrophages in dual-chamber co-culture system for 48 hrs at 33°C. Cytoplasmic and nuclear protein was extracted from YAMC cells using NE-PER Nuclear and Cytoplasmic Extraction Reagents (ThermoFisher Scientific, Waltham, MA, USA) according to the manufacturer's instructions.

### Western blotting

Whole-cell extracts (20 μg) were separated by SDS-PAGE and blotting performed as previously described [16]. The source and dilution of primary antibodies are listed in Supplementary Table 2. Horseradish peroxidase (HRP)-donkey anti-goat IgG conjugate (Santa Cruz Biotechnology), HRP-goat anti-rabbit IgG conjugate (Life Technologies), and HRP-goat anti-mouse IgG conjugate (Cell Signaling Technology) were used as secondary antibodies. Signals were generated by Clarity^TM^ Western ECL Substrate (Bio-Rad) and captured by ChemiDoc XRS+ system (Bio-Rad).

### Immunohistochemistry (IHC) and immunofluorescent (IF) staining

Epitope retrieval for IHC and IF staining were performed as previously described [16]. Primary antibodies and dilution for IHC and IF staining are shown in Supplementary Table 2. HRP-goat anti-mouse IgG conjugate (Cell Signaling Technology) and HRP-goat anti-rabbit IgG conjugate (Life Technologies) were used as secondary antibodies. Chromogenic color development was performed using 3,3’-diaminobenzidine enhanced liquid substrate (Sigma, St. Louis, MO, USA) and nuclei counterstained by Mayer's hematoxylin (Sigma). IF staining for YAMC cells and mouse colon biopsies was performed as previously described [34]. Goat anti-rabbit IgG (H+L)-Alexa 647 conjugate and goat anti-mouse IgG (H+L)-Alexa 647 conjugate (Life Technologies) were used as secondary antibodies. Nuclei were counterstained by 4’,6-diamidino-2-phenylindole. Images were analyzed by laser scanning confocal microscopy (Leica Microsystems, Buffalo Grove, IL, USA).

### *CTNNB1* silencing

Silencing of β-catenin was performed using small interfering RNA (siRNA) as previously described [34]. Human *CTNNB1*-specific siRNAs were purchased from Cell Signaling Technology and scrambled nontargeting siRNA from GE Dharmacon (Lafayette, CO, USA). Transient transfections were conducted using lipofectamine® 3000 reagent and gene silencing confirmed by qRT-PCR and Western blotting.

### Statistical analysis

Data were expressed as means with standard deviations (SD) shown for at least 3 independent experiments. Student's *t* test was used for comparisons between experimental and control groups. *P* values < 0.05 were considered significant.

## SUPPLEMENTARY MATERIALS FIGURES AND TABLES


