# Supplementary material for: Commensal-infected macrophages induce dedifferentiation and reprogramming of epithelial cells during colorectal carcinogenesis
Source: Oncotarget. 2017 Nov 1;8(60):102176–90. doi: 10.18632/oncotarget.22250 (PMC5731944; doi:10.18632/oncotarget.22250)
Supplement: Supplementary file 1 [file oncotarget-08-102176-s001.pdf]

# Commensal-infected macrophages induce dedifferentiation and reprogramming of epithelial cells during colorectal carcinogenesis

## SUPPLEMENTARY MATERIALS

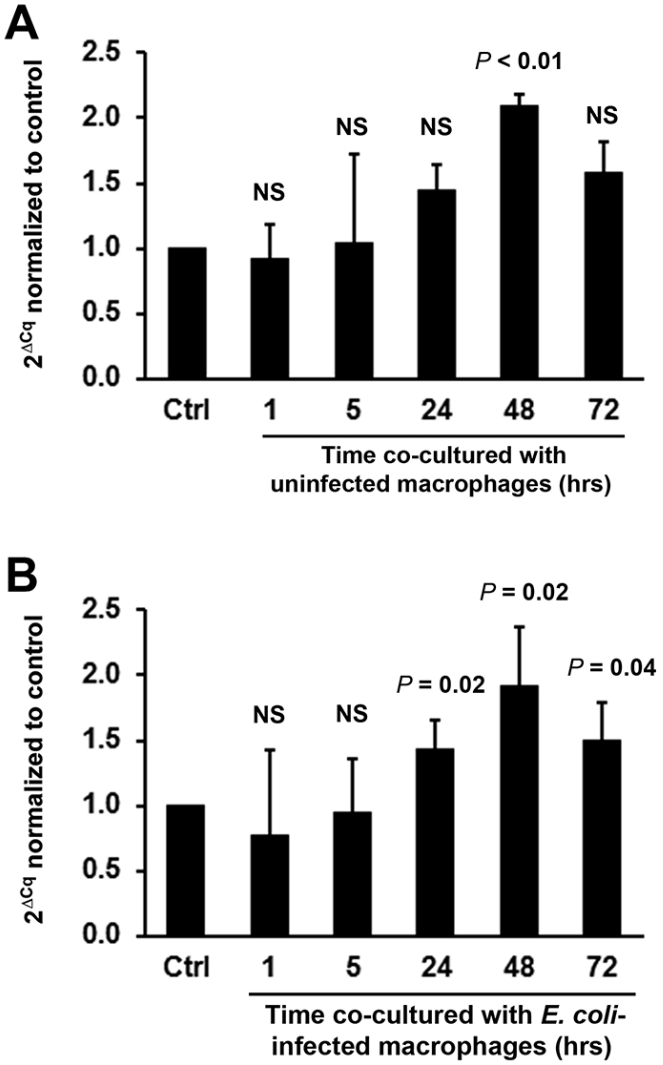


**Supplementary Figure 1: *Ctnnb1* expression in YAMC cells exposed to uninfected and *E. coli*-infected macrophages.** **(A)**, *Ctnnb1* expression in YAMC cells transiently increases after 48 hrs treatment with uninfected macrophages compared to untreated control. **(B)**, *Ctnnb1* expression increases after 24 - 72 hrs treatment with *E. coli* DH5α-infected macrophages compared to untreated control. NS, not significant. Data represent mean ± SD for 3 independent experiments.

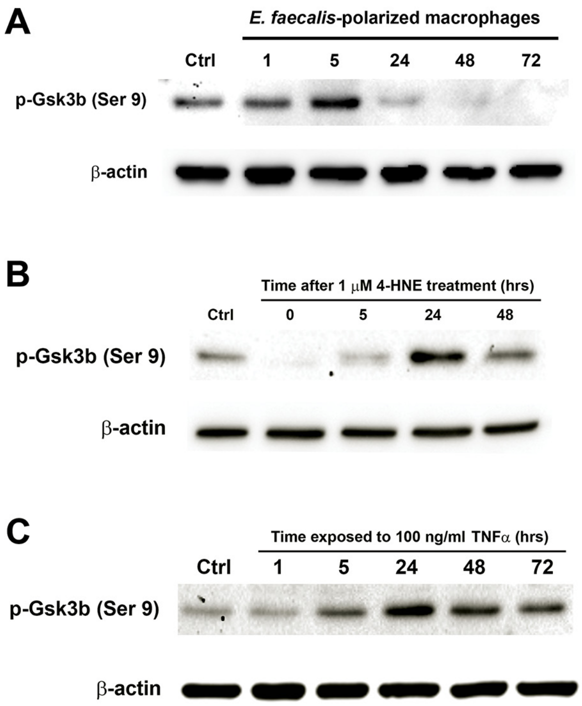


**Supplementary Figure 2: MIBE-induced phosphorylation of Gsk3β (Ser[9]). (A)**, Phosphorylated Gsk3β (Ser[9]) is transiently increased in YAMC cells exposed to *E. faecalis*-infected macrophages for 5 hrs and decreased afterwards. **(B)**, p-Gsk3β (Ser9) is transiently increased 24 hrs after 1 hr treatment with 4-HNE. **(C)**, Increased p-Gsk3β is also noticed for cells treated with TNFα for 24 hrs and subsequently decreased.

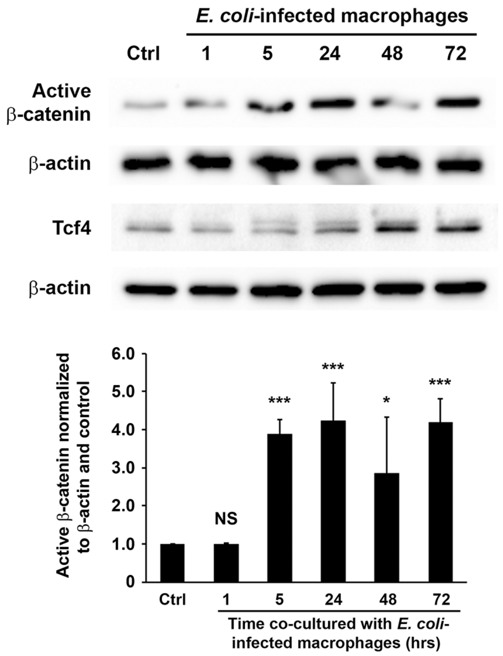


**Supplementary Figure 3: Activation of β-catenin by *E. coli*-infected macrophages.** Western blots show increased active β-catenin and Tcf4 (*upper*) in YAMC cells exposed to *E. coli*-infected macrophages (100 multiplicity of infection) after normalization to β-actin and untreated controls (*lower*). Data represent mean ± SD for 3 independent experiments.

**Supplementary Table 1: Primers for qRT-PCR**

| Genes | Primer sequence (bp) | PCR product |
|---|---|---|
| Human *ACTB* | | 148 |
| forward | 5'- ACCTTCTACAATGAGCTGCG -3' | |
| reverse | 5'- CCTGGATAGCAACGTACATGG -3' | |
| Human *CTNNB1* | | 150 |
| forward | 5'- GTTCAGTTGCTTGTTCGTGC -3' | |
| reverse | 5'- GTTGTGAACATCCCGAGCTAG -3' | |
| Human *DCLK1* | | 118 |
| forward | 5'- AAAGTCTGCAGCTCGATGG -3' | |
| reverse | 5'- CATCTCCTATTGTTCTTCCGACT -3' | |
| Murine *Actb* | | 147 |
| forward | 5'- ACCTTCTACAATGAGCTGCG -3' | |
| reverse | 5'- CTGGATGGCTACGTACATGG -3' | |
| Murine *Ctnnb1* | | 135 |
| forward | 5'- GCTATTCCACGACTAGTTCAGC -3' | |
| reverse | 5'- AGCTCCAGTACACCCTTCTAC -3' | |
| Murine *Dclk1* | | 83 |
| forward | 5'- TGCCGACTGAACTGTATCTTG -3' | |
| Reverse | 5'- TGTGTATTTGCTAGTGGAGGTG -3' | |

**Supplementary Table 2: Primary antibodies and dilution used in this study**

| Application and antigen | Vendor | Catalogue Number | Host | Clonality | Dilution |
|---|---|---|---|---|---|
| *Western bloting* | | | | | |
| Active β-catenin | Cell Signaling Technology | 4270 | rabbit | Poly | 1:1,000 |
| β-actin | Santa Cruz Biotechnology | sc-81178 | Mouse | Mono | 1:2,000 |
| c-Myc | Santa Cruz Biotechnology | sc-40 | mouse | Mono | 1:500 |
| CD44 | Cell Signaling Technology | 5640 | Mouse | Mono | 1:500 |
| Dclk1 | Abgent | AP7219b | Rabbit | Poly | 1:1,000 |
| Klf4 | Millipore | 09-821 | Rabbit | Poly | 1:500 |
| Oct3/4 | Santa Cruz Biotechnology | sc-9081 | Rabbit | Poly | 1:200 |
| p-Gsk3β (Ser$^{9}$) | Santa Cruz Biotechnology | sc-11757 | Goat | Poly | 1:200 |
| Sox2 | Abgent | AP2048D | Rabbit | Poly | 1:1,000 |
| Tcf4 | Santa Cruz Biotechnology | sc-8631 | Goat | Poly | 1:200 |
| Wif1 | Cell Signaling Technology | 2064 | Rabbit | Poly | 1:1,000 |
| Wnt3α | R&D Systems MAB1324 | MAB1324 | Rat | Mono | 1:400 |
| *IHC & IF staining* | | | | | |
| Active β-catenin | Millipore | 05-665 | Mouse | Mono | 1:250 |
| c-Myc | Abgent | AP1985B | rabbit | Poly | 1:25 |
| Dclk1 | Abgent | AP7219b | Rabbit | Poly | 1:50 |
| Klf4 | Millipore | 09-821 | Rabbit | Poly | 1:100 |
| Oct3/4 | Santa Cruz Biotechnology | sc-9081 | Rabbit | Poly | 1:100 |
| Sox2 | Cell Signaling Technology | 3728 | Rabbit | Mono | 1:50 |
